# An integrated 2D framework for quantifying cellular mechanics reveals the impact of juxtacrine Notch signalling on directed collective migration of endothelial cells

**DOI:** 10.1186/s12915-025-02396-4

**Published:** 2025-10-01

**Authors:** Janine Grolleman, Carlijn V. C. Bouten, Vito Conte, Cecilia M. Sahlgren

**Affiliations:** 1 Department of Biomedical Engineering, Soft Tissue Engineering and Mechanobiology, Eindhoven University of Technology, Eindhoven, 5612AE The Netherlands; 2 Institute for Complex Molecular Systems, Eindhoven University of Technology, Eindhoven, 5600MB The Netherlands; 3 Institute for Bioengineering of Catalonia, The Barcelona Institute of Science and Technology, Barcelona, 08036 Spain; 4 Faculty of Science and Engineering, Cell Biology, Åbo Akademi University, Turku, 20520 Finland

**Keywords:** Collective endothelial migration, Migration kinematics, Cell–matrix dynamics, Cell–cell dynamics, Juxtacrine Notch signalling, In vitro framework

## Abstract

**Background:**

Collective migration is the coordinated movement of a group of cells—a fundamental process in health and disease. Many models have been developed to study the molecular and physical mechanisms of collective migration. However, the aim of this study is to engineer a flexible in vitro framework that allows for mechanobiological quantification of the separate and combined contributions of individual cell mechanics to the directed migration of a collective. We utilised this framework to understand the role of juxtacrine Notch signalling during collective endothelial migration—an essential process during the formation of new blood vessels (known as angiogenesis).

**Results:**

This framework enables users to perform high spatiotemporal analysis of migrative behaviour, cell–matrix traction forces, and intercellular forces in different microenvironments. With this framework, we show that Notch inhibited collectives adopt a distinct regime of directed collective migration. Whereas the directionality of migration, traction forces and intercellular forces are not affected by Notch inhibition, we observed spatiotemporal differences in migration speed, traction force magnitude and normal and shear stresses within Notch-inhibited collectives.

**Conclusions:**

The in vitro framework is a powerful approach for dissecting the mechanisms of collective migration. With this framework, we show that a potential link exists between the juxtacrine signalling of Notch and an increased mechanical cohesiveness among collective cells.

**Supplementary Information:**

The online version contains supplementary material available at 10.1186/s12915-025-02396-4.

## Background

The coordinated movement of a group of cells, known as collective cell migration, is a fundamental process that plays pivotal roles in various physiological and pathological phenomena [1–5]. This process is driven by cell and tissue mechanics at the supracellular scale [6–8]. Several biological models have been proposed and used to study the molecular and physical mechanisms of collective cell migration both in vivo and in vitro. In vivo models have allowed the combination of genetics and pharmacological treatments in a physiological context, but their complex architecture and the interplay of different processes constrain mechanobiological quantification [5, 9]. In contrast, in vitro models have been designed as simplified mimics of the complex in vivo architecture, thus providing higher experimental control and allowing for more extensive mechanobiological quantification*.*

Among in vitro models, 3D implementations offer the possibility of achieving higher levels of hierarchical biological organisation and architecture to better mimic in vivo processes. Still, mechanobiological quantification is inherently more complex [6, 7]. On the other hand, 2D in vitro models of collective migration are more widely used because they offer an optimal compromise between their ability to mimic specific aspects of in vivo processes and maximising the use of techniques for mechanobiological quantification. A significant feature behind the widespread use of 2D in vitro models to investigate the mechanobiology of collective cell migration—and, more generally, the mechanics of cells—is their ability to systematically and controllably mimic both the compliance and the organisation of the extracellular matrix (ECM). In such 2D models, ECM is emulated via deformable hydrogel substrates where substrate stiffness accounts for ECM compliance, and the micropatterning of protein coatings for selective cell attachment on top of the hydrogel substrates can emulate ECM organisation, density, and topology. Protein micropatterning on hydrogel substrates has been previously extensively utilised to confine the motion of a single cell [10, 11] or that of a cellular monolayer [10, 12–14], as well as to direct collective cell migration on straight [15, 16] or circular strips [17, 18], or to emulate the presence of obstacles against collective migration [19].

Here, we integrated these approaches into an in vitro framework to enable a structured comparative study of four distinct fundamental migration modes. Using this experimental framework, we systematically quantified the separate and combined contributions of individual cell mechanics to directed collective migration in human umbilical vein endothelial cells (HUVECs). During angiogenesis, endothelial cells collectively invade the surrounding ECM in a directed manner [6–8, 20], steered by chemoattractants [21]. Angiogenesis is a complex process coordinated by the juxtacrine Notch signalling pathway [22]. By employing this in vitro framework, we found that Notch signalling enables HUVEC collectives to acquire significant speed differentials only at the expense of generating increased forces both at the interface with the underlying matrix and other cells of the collective. This characteristic is lost in endothelial cells where Notch signalling is inhibited, as these collectives can still migrate directionally and significantly vary their migration speed without acquiring substantial force differentials at the cell–cell and cell–matrix interfaces.

## Results

### Designing and building an in vitro framework to systematically quantify the separate and combined contributions of individual cell mechanics to adhesion-based directed collective cell migration in 2D

Directed collective migration of cells—based on the formation of focal adhesions at the interface between cells and a flat substrate—involves the coordination of several mechanical components. Firstly, individual cells must adhere to the matrix and exert forces (adhesion-based migration), either to remain in place or to move towards free space or move along neighbouring cells through intercellular cooperation. Secondly, the entire cohort of cells must collectively invade the surrounding free space and must do so in a preferential direction.

**Fig. 1:**
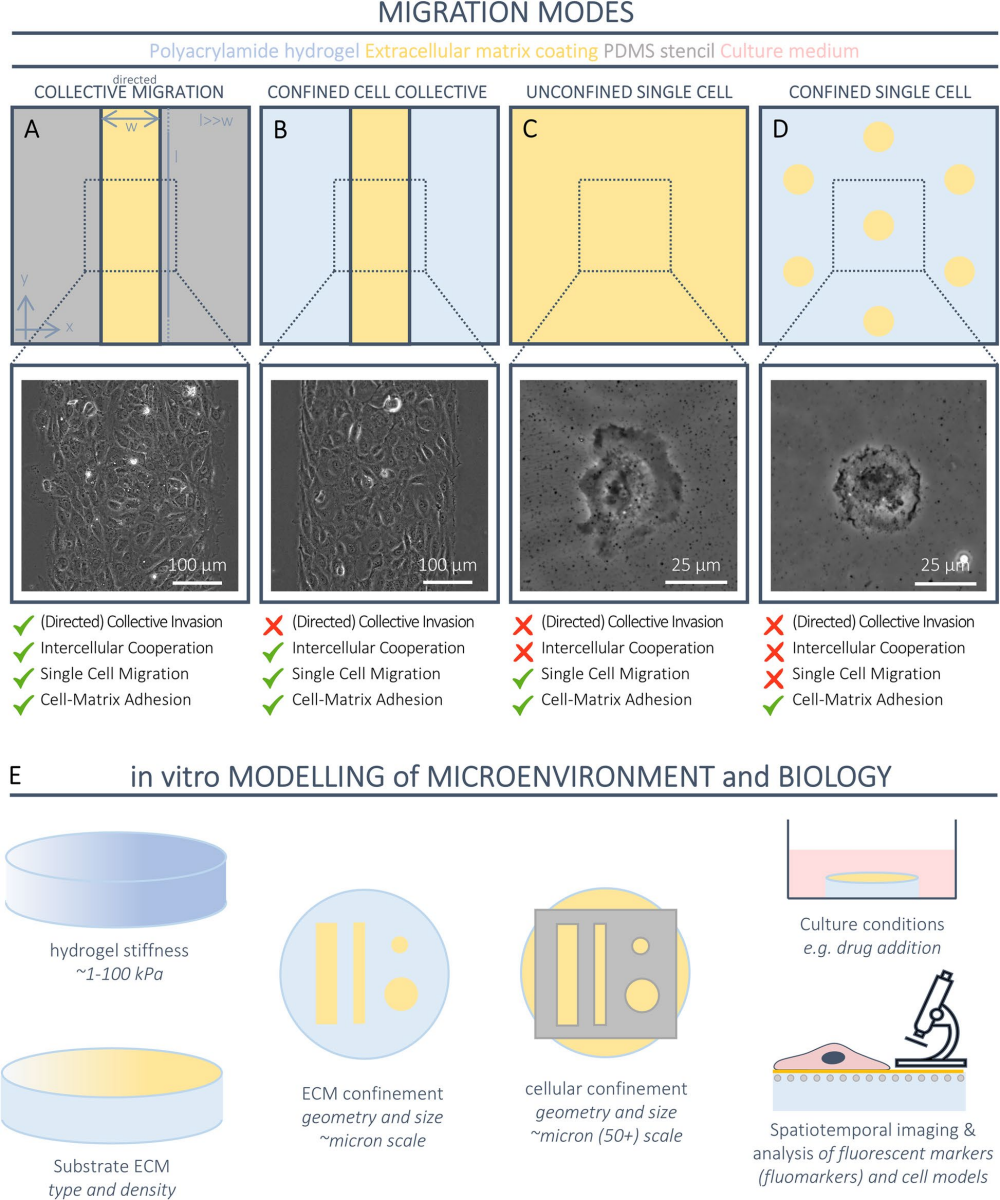
An in vitro framework for the systematic study of collective migration. **A–D** Four different migration modes were modelled based on the principles of directed collective invasion, intercellular cooperation, single-cell migration, and cell–matrix adhesion. A schematic representation and phase contrast images of an unconfined cell collective at the start of directed migration (**A**), a confined cell collective (**B**), an unconfined single cell (**C**), and a confined single cell (**D**). **E** A schematic overview of the possibilities to tune the microenvironment and biology within the migration framework

With that in mind, we set out to create a 2D in vitro framework to analyse the separate and combined contributions of those mechanical components to directed collective migration (Fig. 1A–D). To this end, we developed tools to systematically image and mechanically quantify four adhesion-based migration modes: (i) cellular collectives undergoing “directed collective migration” (Fig. 1A), to study the extent to which the global motion is in a preferential direction when starting from the same initial condition of collective confinement utilised for controlled repeatability; (ii) “confined cell collectives” (Fig. 1B), to study the effects of restricting directed collective migration without affecting intercellular cooperation or individual cell migration; (iii) “unconfined single cells” (Fig. 1C), to investigate the effects of inhibiting collective migration and intercellular cooperation while still preserving individual cellular migration; and (iv) “confined single cells” (Fig. 1D), to examine the effects of inhibiting collective migration, intercellular cooperation, and individual cell migration while retaining the ability of cells to adhere to the substrate and exert force. Integrating these four modes of migration into a unified framework enables systematic comparisons of distinct cellular behaviours at both individual and collective levels, offering a comprehensive understanding of how each mode—separately and together—contributes to the mechanics of directed collective migration. Furthermore, this framework can be implemented in a multi-well plate approach, offering the possibility to tightly control experimental variables—such as inter-experimental variability, stiffness, cell seeding, cell culture, and imaging conditions—thus reducing potential noise when comparing experiments.

As for creating confined cellular cohorts undergoing directed collective migration systematically and controllably, we placed a polydimethylsiloxane (PDMS) stencil with a rectangular opening on top of a uniformly ECM-coated polyacrylamide (PAA) hydrogel (Fig. 1A, Additional file 1: Fig. S1). After seeding cells, the PDMS stencil is removed and cells are free to invade the open space surrounding the collective, thus emulating directed collective migration [23–27]. To boost migration along a preferential axis, it is sufficient to design the rectangular opening with its length (along the *y*-axis in our case) significantly greater than its width (along the *x*-axis), therefore promoting the predominant movement of the cellular collective in the *x*-axis direction (Fig. 1A, Additional file 2: Video S1,2). As for systematically creating cellular cohorts that are permanently confined, we restricted the ECM-coating of PAA hydrogels to predesigned domains of interest so that cells were unable to invade the open space due to the absence of ECM outside these domains (Fig. 1B, Additional file 1: Fig. S1, Additional file 3: Video S3,4) [28, 29]. This method was also employed to confine single cells on the hydrogel substrates (Fig. 1D, Additional file 1: Fig. S1, Additional file 4: Video S5,6), whereas we uniformly coated the entire surface of the hydrogels and adopted low cell seeding densities to obtain single cells in a state of free migration (Fig. 1C, Additional file 1: Fig. S1, Additional file 5: Video S7,8).

The migration substrates utilised in the framework are designed to emulate a swatch of stroma microenvironments. Specifically, these substrates allow the systematic modulation of mechanical and chemical cues from the cellular microenvironment, including matrix stiffness, matrix composition and density, and paracrine signalling (via pharmaceutical conditioning of the cell medium). Importantly, the framework allows the monitoring of these parameters in conditions of longer live spatiotemporal imaging (Fig. 1E). The modulation of these experimental parameters within each migration mode enables the study of directed collective cell migration under varying biological conditions. This flexibility in adjusting experimental conditions, such as tuning matrix stiffness, cell culture environment, and pharmacological interventions, allows the accommodation of a wide range of experimental setups, extending beyond the specific focus on angiogenesis.

**Fig. 2:**
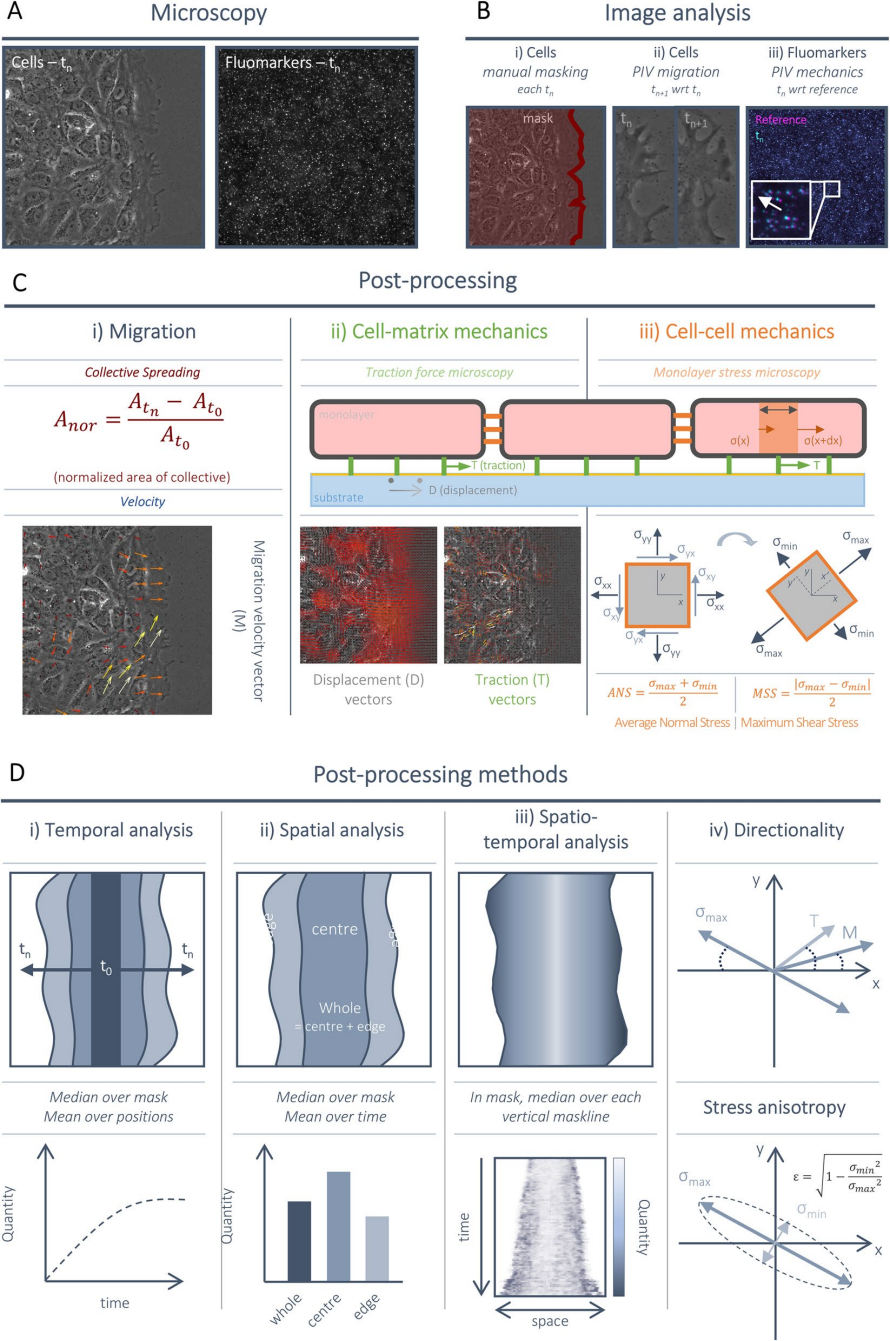
Collective migration mechanical analysis pipeline. **A** Microscopy was used for timelapse phase contrast imaging of the cells and fluorescence imaging of the fluomarkers within the polyacrylamide (PAA) hydrogel. **B,C** Image analysis (**B**) and post-processing (**C**) were performed on each position for each timepoint. A manual mask of the cell collective was defined (**B**) and used as a measure of collective spreading (**C**) and for relevant data retrieval (**D**). Cellular migration was measured by performing Particle Image Velocimetry (PIV) on two consecutive phase contrast images in time (**B**) resulting in a vectorial field of migration velocity vectors (**C**). Fluomarkers displacements were measured by performing PIV on a timelapse fluomarkers image compared to the reference image (**B**), resulting in a displacement vectorial field (**C**). Cellular tractions were computed from the displacement by using traction force microscopy (**C**). Intercellular stresses were computed using monolayer stress microscopy (**C**), the maximum and minimum principal stresses were computed, and the average normal stress (ANS) and maximum shear stress (MSS) is calculated (**C**). **D** Post-processing was performed using different methods and is explained extensively in the Methods section. Temporal analysis was performed by calculating the mean value across multiple positions for each timepoint. Spatial analysis was performed by dividing the mask into regions, and calculating, for each position, the mean value over multiple timepoints. Spatiotemporal analysis was performed by generating a kymograph, where the x-direction represents space on the horizontal axis, and time is plotted on the vertical axis. Directionality of a quantity is determined by the angle with respect to the migration direction (*x*-axis). Stress anisotropy was calculated by calculating the eccentricity of the ellipse plotted on top of the maximum and minimum principal stresses

To analyse cellular mechanics during collective migration, we designed and built an automated pipeline that couples the in vitro framework with a combination of different microscopy techniques (Fig. 2A,B) and quantification methods to measure cellular kinematics [23, 30, 31] along with the cellular forces at the cell–matrix [23, 32, 33] and inter/intra-cellular levels [34, 35] (Fig. 2C) both over space and time (Fig. 2D).

### Juxtacrine Notch signalling regulates collective cellular speed but not directionality in endothelial cell monolayers free to migrate

**Fig. 3:**
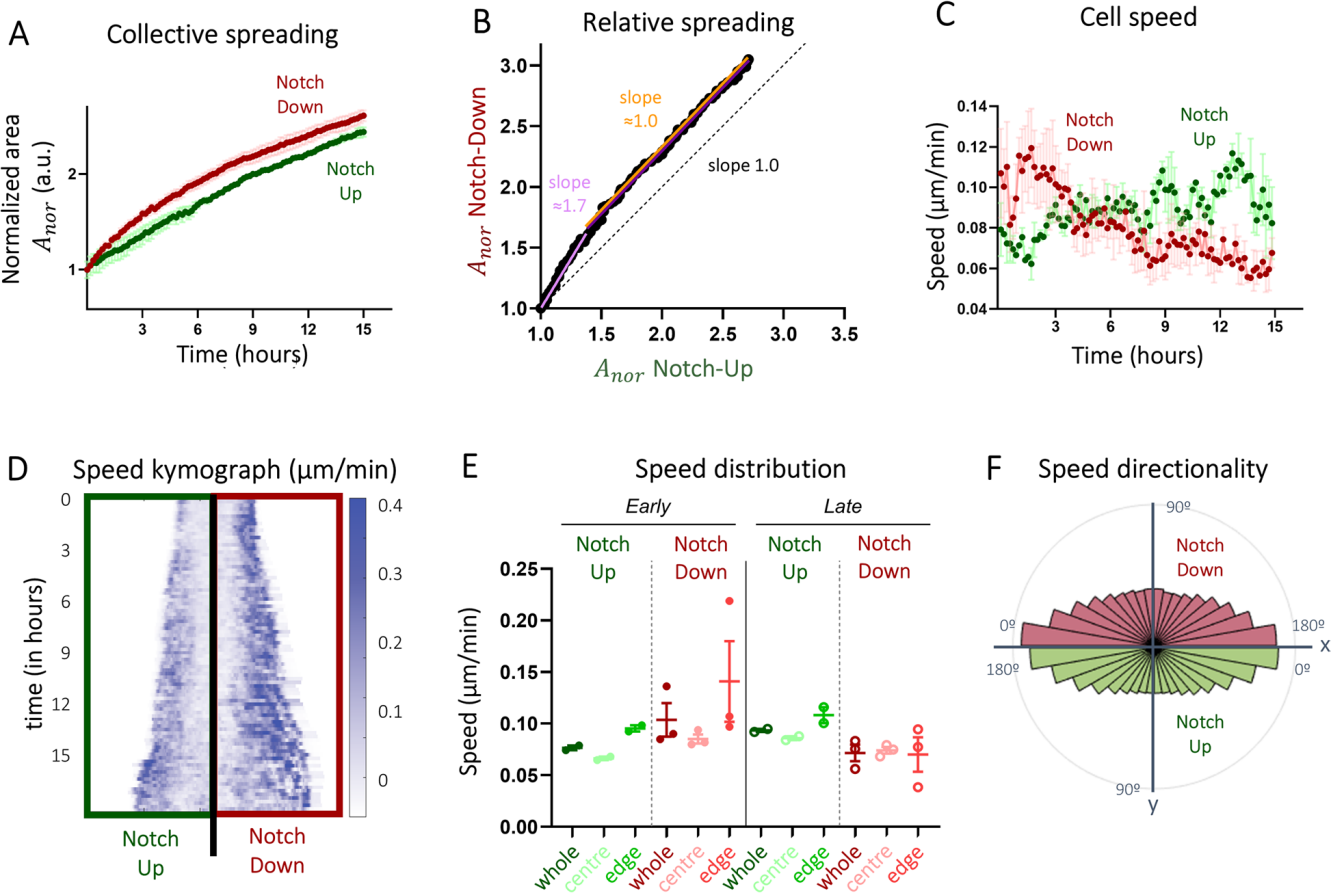
Inhibition of cell–cell communication affects migration speed but not directionality during directed collective migration (Fig. 1A).** A** Collective spreading was quantified as the normalised increase in the area of the collective mask over time, and the data were plotted on a logarithmic scale. Each dot represents one timepoint, which is an average value over multiple positions. **B** Relative spreading is the normalised area of Notch-Up collectives plotted against the normalised area of Notch-Down collectives over time. Each dot is the average value of multiple positions. Slope of the graph is computed using linear regression. The slope was calculated separately for early timepoints (*t* < 4 h, purple) and late timepoints (*t* > 4 h; orange). **C** Time evolution of cell speed within the collective based on PIV analysis of phase contrast images. Each dot represents one timepoint, which is an average value over multiple positions. **D** A representative (half) kymograph of cell speed with space on the horizontal axis and time on the vertical axis. **E** Cell speed distribution analysis of the central zone and the edge zone of the collective compared to the whole collective during early phase of migration (*t* < 4 h; left) and late phase of migration (*t* > 4 h; right). Each dot represents one position, which is an average value over the specified time and the specified space. **F** Speed directionality analysis as a measure of the angle of the migration velocity vector with respect to the *x*-axis. Bars represent an angle range of 10° and values are absolute (0–180° instead of − 180–180°). Notch-Up represents collectives treated with DMSO; Notch-Down represents collectives treated with DAPT. Data is represented as mean ± SEM

Next, we applied our framework to examine how individual cell mechanics contribute — both separately and together — to directed collective migration in untreated endothelial cells (ECs) and in ECs treated with a Notch signalling inhibitor. To model the physiological stiffness of the human vessel stroma underlying the vascular endothelium, we engineered PAA hydrogel substrates for the framework (Fig. 1E) to have a stiffness of 12 kPa [36]. To mimic the ECM protein content of the vascular system, we coated the hydrogel substrates with collagen type IV (Fig. 1E)—an ECM protein that is found in the endothelial basement membrane and that is known to promote angiogenesis [37, 38]. Using a PDMS stencil, we micropatterned HUVECs in rectangles of 300 µm in width (along the *x*-axis) and 2.5 cm in length (along the *y*-axis)—therefore ensuring directed collective migration in the *x*-axis (Fig. 1A,E). Juxtacrine signalling through Notch is active in EC collectives [39, 40], where it has been shown to affect their migratory behaviour [6, 41]. To this end, similarly to previous studies [42, 43], we inhibited Notch signalling in endothelial cells by adding the ϒ-secretase inhibitor DAPT to the culture medium via a DMSO vehicle (hereafter referred to as the “Notch-Down” condition). In comparison, endothelial cells cultured in medium containing only the vehicle DMSO and spontaneously having high Notch activity were used as controls (hereafter referred to as the “Notch-Up” condition). Endothelial cells were pre-treated overnight in medium containing either DMSO only (Notch-Up) or DAPT and DMSO (Notch-Down). The PDMS stencil was removed at *t* = 0 h, the medium was refreshed without altering its composition, and the cell collectives were left free to invade the free space surrounding them while being live-imaged for 15 h with a time interval of 10 min. Over the span of 15 h, Notch-Up EC collectives spread up to 2.7 times their initial area at *t* = 0 h, whereas Notch-Down EC collectives spread up to 3.1 times (Fig. 3A). Interestingly, the Notch-Down EC collectives spread almost twice as fast as Notch-Up ECs during the first 4 h (slope 1.7) before the two cell types stabilised to similar speeds at later stages (slope 1.0; Fig. 3B). Next, we looked at velocity of migration at the local level of single ECs within the collectives by means of particle image velocimetry (PIV) on consecutive phase-contrast images (Fig. 2B,ii). This provided vectorial fields of cell velocities in time (Fig. 2C,i), with the magnitude of velocity vectors henceforth referred to as cellular “speed” and the angle of these vectors form with the *x*-axis as cellular “directionality”. Speed analyses showed that Notch-Down ECs were in average faster within the collective than Notch-Up ECs at early stages of migration (*t* < 4 h, Fig. 3C), in line with the observed increased spreading of the collective as a whole (Fig. 3A). Interestingly, this behaviour did not depend on the monolayer being able to collectively invade or not (Fig. 1B), as shown in the case of the entire EC collective being kept confined to the initial domain at *t* = 0 h (Additional file 1: Fig. S2). However, Notch-Down EC collectives surprisingly became slower than Notch-Up EC collectives at later time points (*t* > 4 h, Fig. 3C).

To average out spatial–temporal fluctuations of the cellular velocity field and visualise velocity patterns in our collectives, we averaged the migration speed in space along lines concentric to the edge of the collective (migration front, Fig. 2D). Upon displaying these space averages as a function of distance from the endothelial domain’s edge (Fig. 2D, iii), we obtained kymographs of the net cellular speed of cells within the collective (Fig. 3D). We observed a tendentially higher migration speed at the edge of both Notch-Up and Noth-Down EC collectives compared to their central domains for *t* < 4 h (Fig. 3D). To better determine these spatial trends of migration speed and account for variability across samples, we further restricted quantifications to either the central or edge domains of EC collectives (Fig. 2D, ii)—in doing so, we made sure that the two domains had areas of the same order of magnitude to ensure that analyses on mechanical quantities would be conducted on statistical populations of similar size. This analysis further confirmed that ECs within both Notch-Up and Notch-Down EC collectives tended to have higher migration speeds at the collective’s edge than at their centres for time points *t* < 4 h (earlier stages), specifically 1.4-fold higher for Notch-Up EC collectives and 1.7-fold higher for Notch-down ones (Fig. 3E). For *t* > 4 h (later stages), however, ECs in Notch-Up EC collectives kept speeds higher at the collective’s edges than centres (1.3-fold higher) whereas speeds of Noth-Down EC collectives at the collective’s edges went down to the average values observed in these collective’s centres (Fig. 3E). Average migration speed within the whole Notch-Up EC collectives, instead, tended to increase by approximately 1.2-fold at later stages compared to earlier ones (Fig. 3E) whereas average migration speed within the whole of Notch-Down EC collectives tended to decrease by 1.4-fold at later stages compared to earlier ones (Fig. 3E).

Finally, we quantified directionality of cell migration velocities within the collectives (Fig. 2D, iv) and observed that ECs in average tended to migrate perpendicularly to the collective’s edge in both the Notch-Up and Notch-Down cases (Fig. 3F). Taken together, our data showed that juxtacrine Notch signalling in EC collectives affects cellular speed but not directionality, in line with previous studies reporting that speed and directionality of cell migration are processes that have distinct dynamics [44]. Since previous studies have also shown that adhesion-based cellular motion relies on physical forces being generated by cells and transmitted to the substrate [45–47], we reasoned that other mechanical quantities related to force might mediate the differences in kinematic behaviours observed thus far between Notch-Up and Notch-Down EC collectives.

### Directed collective migration with active Notch signalling requires higher cellular traction forces than migration under signalling inhibition across comparable distances

**Fig. 4:**
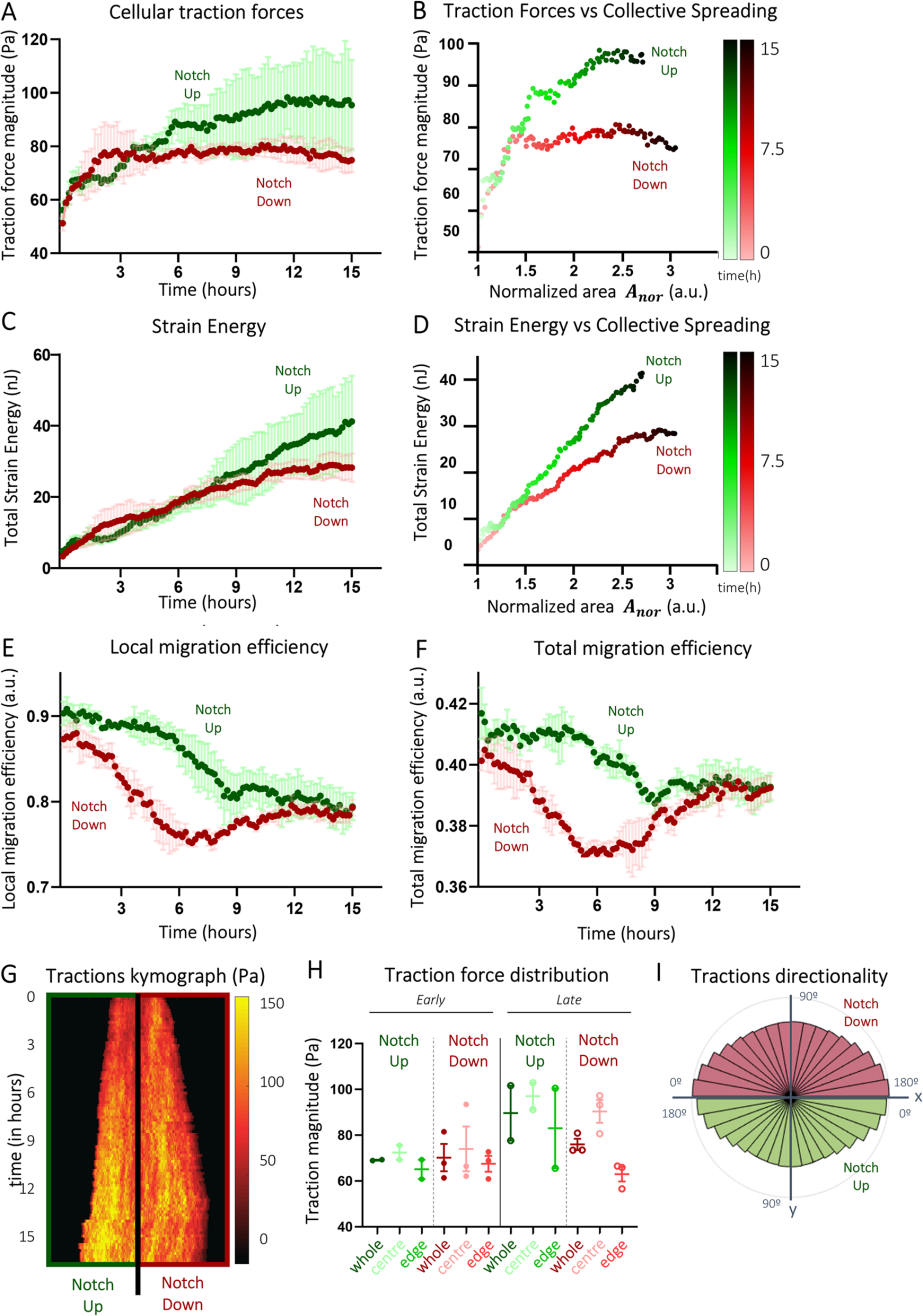
Inhibition of cell–cell communication affects cell–matrix mechanics during directed collective migration (Fig. 1A).** A** Time evolution of traction magnitude within the collective based on PIV analysis of fluomarkers in the hydrogel. Each dot represents one timepoint, which is an average value over multiple positions. **B** Correlation of traction forces and collective spreading in time. Each dot represents one timepoint, which is an average value over multiple positions. **C** Strain energy as a measure of the total energy transferred by the collective to the underlying substrate in time. Each dot represents one timepoint, which is an average value over multiple positions. **D** Correlation of strain energy and collective spreading in time. Each dot represents one timepoint, which is an average value over multiple positions. **E–F** Local and total migration efficiency, showing the alignment between the traction and displacement vectors at a specific location (**E**) or the overall coordination of the collective (**F**). **G** A representative (half) kymograph of traction magnitude with space on the horizontal axis and time on the vertical axis. **H** Traction magnitude distribution analysis of the central zone and the edge zone of the collective compared to the whole collective during early phase of migration (*t* < 4 h; left) and late phase of migration (*t* > 4 h; right). Each dot represents one position, which is an average value over the specified time and the specified space. **I** Tractions directionality analysis as a measure of the angle of the traction vector with respect to the *x*-axis. Bars represent an angle range of 10° and values are absolute (0–180° instead of − 180–180°). Notch-Up represents collectives treated with DMSO; Notch-Down represents collectives treated with DAPT. Data is represented as mean ± SEM

Single and collective cell migration based on adhesion to the ECM relies on cells generating physical forces to set them in motion [45–47]. This is only possible if cellular forces are transmitted from within the cell where they are generated to the underlying matrix via focal adhesions at the cell–matrix interface (traction forces) and to the other cells via cell–cell junctions (intercellular forces). We resorted to Traction Force Microscopy to compute traction-force vector fields (Fig. 2C, ii) driving migration within Notch-Up and Notch-Down EC collectives [23]. Our mechanical analyses showed that average traction forces exerted by ECs in both Notch-Up and Notch-Down EC collectives monotonically increased in a similar fashion during the first 4 h, their magnitude increasing by approximately 1.3-fold around *t* = 4 h (Fig. 4A). While average tractions in Notch-Down EC collectives plateaued at *t* = 4 h, average tractions in Notch-Up EC collectives further increased by 1.2-fold in magnitude before starting plateauing at approximately *t* = 12 h (Fig. 4A). Upon plotting average traction forces at each time point versus the corresponding average state of collective spreading at the same time point (Fig. 4B), we noticed that the traction forces required to expand the collective by 1.5 times in the early stages of migration were approximately similar in the Notch-Up and Notch-Down EC collectives. However, with the passing of time, the tractions required for collectives to expand up to 2.5 times their original span in the later stages of migration were approximately constant for Notch-Down EC collectives whereas an increase of approximately 25% was observed in Notch-Up EC collectives (Fig. 4B). The higher strength of Notch-Up ECs during directed migration also reflected in the higher elastic strain energy transferred by these collective to the underlying substrate compared to Notch-Down EC collectives (Fig. 4C,D). We investigated migration efficiency by measuring the alignment between cellular traction forces and respective hydrogel-substrate displacements, with local efficiency assessing alignment at specific locations (Fig. 4E) and total efficiency reflecting the overall coordination of the collective (Fig. 4F). A value of 1 indicates perfect alignment for optimal directed migration. Notch-Up collectives exhibited higher alignment and sustained greater efficiency early on, before both conditions converged to similar levels at a later time point (Fig. 4E,F). Notably, the differences in traction force magnitude observed during the migration of Notch-Up and Notch-Down EC collectives were not observed during the migration of individual Notch-Up and Notch-Down ECs (Fig. 1C), indicating that differences depended on intercellular cooperation within the collectives (Additional file 1: Fig. S3A). To determine whether these differences also varied within different subdomains of the collectives, we further computed spatiotemporal kymographs of traction magnitudes (Fig. 4G) and quantified average traction magnitude of cells within the edge and central domains of EC collectives (Fig. 4H). We found that there were no substantial differences between traction force magnitudes within the centre and edge domains of Notch-Up EC collectives at time points earlier or later than *t* = 4 h (Fig. 4G,H), nor were there substantial differences for Notch-Down EC collectives in the same time window. However, at later stages (*t* > 4 h), traction force magnitude in the centre of Notch-Down EC collectives tended to increase in average by 1.4-fold (Fig. 4G,H) compared to the collectives’ edges (tractions there approximately remained at the average magnitude levels shown at earlier time points *t* < 4 h)—these differences, however, were not statistically significant (Methods).

Directionality analysis (Fig. 4I) further showed that traction forces were exerted mainly perpendicularly to the migrating edge of both Notch-Up and Notch-Down EC collectives—thus acting as propelling forces for directed collective migration. This was in line with our previous results showing that Notch-signalling had no significant effects on migration directionality in both Noth-Up and Notch-Down EC collectives (Fig. 3F), contrary to what was observed for cell migration speeds within the collective (Fig. 3C,E). Thus, we further investigated whether changes in migration speed in Notch-Up and Noth-Down EC collectives correlated with traction magnitudes in a different way for the two types of EC collectives. We found that higher traction magnitudes correlated with higher migration speeds within Notch-Up EC collectives both in early and later stages of directed collective migration, indicating that higher traction magnitudes were required for these collectives to migrate at higher speeds at later stages compared to earlier ones (Additional File 1: Fig. S3B). This was not the case for Notch-Down EC collectives, where higher migration speeds could be achieved via smaller differentials in traction force magnitude compared to the case of Notch-Up EC collectives (Additional File 1: Fig. S3B).

To investigate whether this correlation depended on the cooperative behaviour among ECs of the collectives, we studied the relationship between traction magnitudes and migration speeds for single ECs that are free to move (Fig. 1C) compared to single ECs that are confined (Fig. 1D). Our analyses showed no correlation between traction force magnitude and migration speed of single ECs (Additional File 1: Fig. S3C), thus indicating that the differences we observed between the traction-speed relationships of Notch-Up and Notch-Down EC collectives were due to intercellular cooperation within the collective. Previous studies reported that cooperative force transmission between cells (intercellular stresses) during collective cell migration can affect migration speed and directionality via a phenomenon referred to as Plithotaxis [34, 48–50]. Thus, we turned our attention to mechanical stresses within EC collectives in the Notch-Up and Notch-Down conditions.

### ECs with high and low Notch signalling undergo distinct regimes of directed collective migration regulated by Plithotaxis

**Fig. 5:**
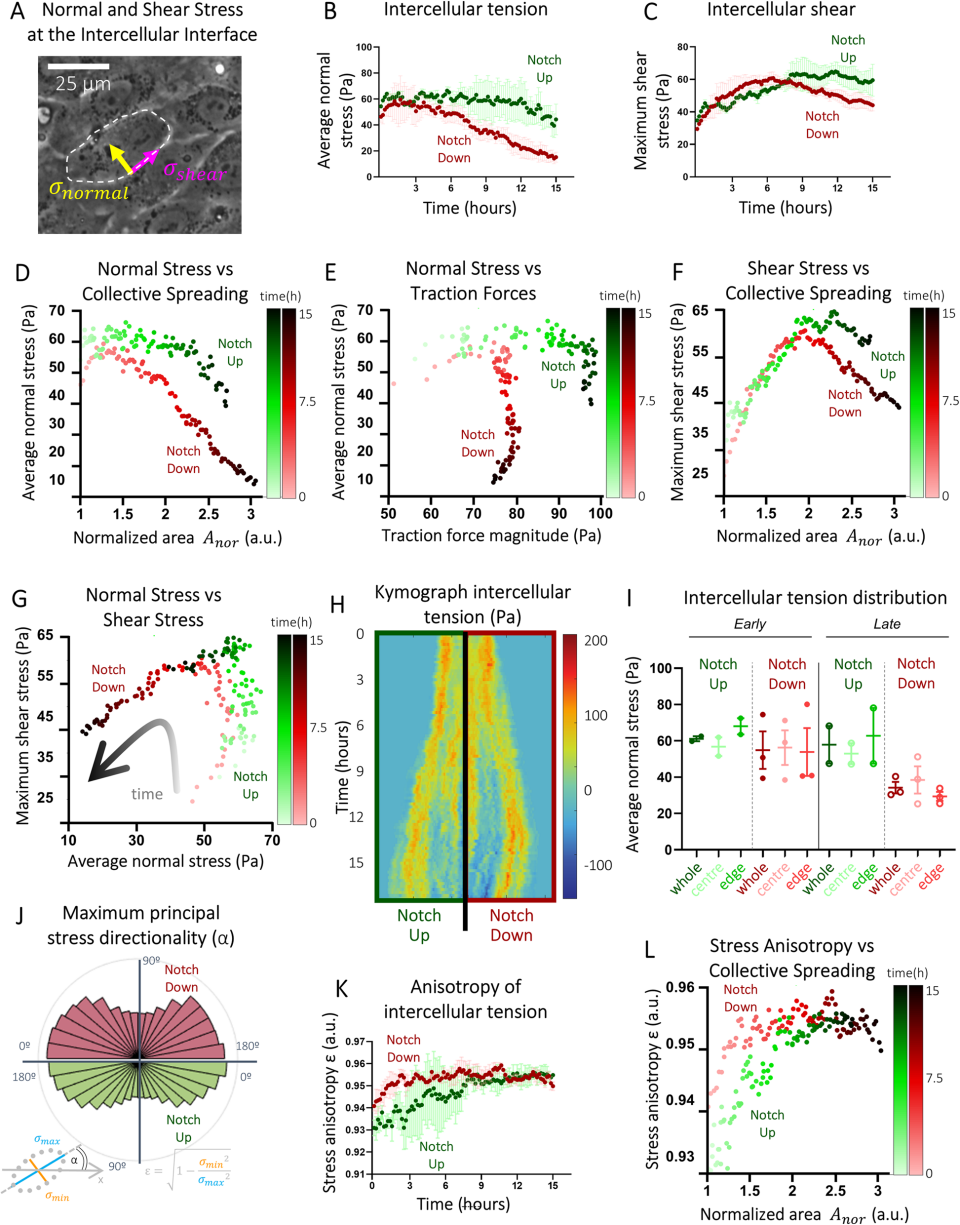
Inhibition of cell–cell communication affects cell–cell mechanics during directed collective migration (Fig. 1A). **A** In a cellular collective, mechanical stresses are exerted normally (negative compression and positive tension) or tangentially to junctional surfaces (49). **B,C** Time evolution of the average normal stress (**B**), as a measure of intercellular tension, and maximum shear stress (**C**), as a measure of intercellular shear, within the collective. Each dot represents one timepoint, which is an average value over multiple positions. **D–G** Correlation plots in time of normal stresses and collective spreading (**D**), normal stresses and traction forces (**E**), shear stresses and collective spreading (**F**), and normal stresses and shear stresses (**G**). Each dot represents one timepoint, which is an average value over multiple positions. **H** A representative (half) kymograph of average normal stresses with space on the horizontal axis and time on the vertical axis. **I** Average normal stress distribution analysis of the central zone and the edge zone of the collective compared to the whole collective during early (*t* < 4 h; left) and late migration (*t* > 4 h; right). Each dot represents one position, which is an average value over the specified time and the specified space. **J** Maximum principal stress directionality analysis as a measure of the angle of the maximum principal stress with respect to the *x*-axis. Bars represent an angle range of 10° and values are between 0° and 180°. **K** Anisotropy of the intercellular tension indicated as the eccentricity of the ellipse plotted on the maximum and minimum principal stresses. Each dot represents one timepoint, which is an average value over multiple positions. **L** Correlation plot in time of stress anisotropy and collective spreading. Each dot represents one timepoint, which is an average value over multiple positions. Notch-Up represents collectives treated with DMSO; Notch-Down represents collectives treated with DAPT. Data is represented as mean ± SEM

In a cellular collective, mechanical compression and tension are stresses exerted normally to the junctional surface of cells (negative values represent compression and positive ones tension), whereas shear stress is exerted tangentially to junctional surfaces [49] (Fig. 5A). To compute inter- and intra-cellular mechanical stresses within the EC collectives, we resorted to Monolayer Stress Microscopy (Fig. 2C, iii) [34, 35]. Specifically, we computed the time evolution of the average normal stress (Fig. 5B) and the maximum shear stress (Fig. 5C) locally within the EC collective—the maximum shear stress can be utilised as a proxy for how strongly ECs are sliding with respect to each other at their mutual interface, whereas average normal stress gauges the amount of tension or compression that ECs are subject to within the collective (Fig. 5A).

Our mechanical analyses showed that both Notch-Up and Notch-Down EC collectives were immediately under tension while expanding (Fig. 5B,D) and generating higher traction forces (Figs. 5E and 4A,B), as shown by average normal stress having positive values. Notch-Up EC collectives were able to expand under small differentials of tension for about 12 h—average intercellular tension was approximately constant around 60 Pa during this interval (Fig. 5B,D). During the same time interval, Notch-Up EC monolayers reached 2.5 times their original span (Fig. 3A) with intercellular shear stress also steadily increasing up to average values comparable with those of intercellular tension (Fig. 5C,F). For *t* > 12 h, Notch-Up EC collectives kept expanding with higher differentials of decreasing intercellular tension and shear (Fig. 5B–D,F) and traction values decreasing by approximately 30% (Fig. 5E). Comparatively, Notch-Down EC collectives were able to initially sustain expansion under small differentials of intercellular tension for only about 6 h (half the time of the Notch-Up EC collectives), before continuing expanding with higher differentials of decreasing intercellular tension and shear (Fig. 5B–D,F) as well as traction forces (Fig. 5E). This difference in behaviour between Notch-Up and Notch-Down EC collectives over the time window of our analyses was also clearly highlighted when mapping maximum shear stress against the average normal stress in both cases (Fig. 5G). Notch-Up EC monolayers were able to expand for around 12 h by keeping average intercellular tension at approximately 60 Pa while monotonically increasing intercellular shear between cells by 85% from average levels of approximately 40 Pa (Fig. 5G)—this was followed by a continued expansion of the collective with a reduction in intercellular tension by approximately 40% but a smaller decrease in intercellular shear of only about 10% from *t* = 12 h onwards (Fig. 5G). In contrast, Notch-Down EC collectives could expand under similar levels of average intercellular tension as in Notch-Up EC monolayers for only about 6 h (half the time interval observed in the Notch-Up case), with shear between cells increasing by a similar percentage to that observed in the Notch-Up ECs (Fig. 5G). After *t* = 6 h, the expansion of the Notch-Down EC monolayers continued with a more marked decrease in intercellular tension and shear—nearly twice the reduction seen in the Notch-Up case (70% decrease for average normal stress and a 25% for maximum shear stress).

Next, we further looked into whether the differences in mechanical stresses observed between the Notch-Up and Notch-Down cases were primarily arising from the edge or the central domains of the EC collectives. Spatiotemporal kymographs (Fig. 2D, iii) of average normal stress consistently showed regions of compression at later time stages in Notch-Down EC collectives (Fig. 5H). To better determine these spatial trends and account for variability across samples, we further conducted a zonal analysis (Fig. 2D, ii) for the average normal stress over domains of the EC collectives. We found that intercellular tension at the leading edge of Notch-Up EC collectives tended to be in average 1.2-fold higher than that in central domains both at earlier and later stages (Fig. 5I). Conversely, while no significant differences in average intercellular tension were observed between edge and centre at earlier time points of Notch-Down EC collectives, average values in central domains tended to be 1.3-fold higher than those at the edge (Fig. 5I)—these differences, however, were not statistically significant (Methods).

Having mapped the complete stress distributions within the collectives (Fig. 5A–I), we computed the directions of maximum and minimum normal stresses while intercellular shear is locally zero along these directions (Fig. 2C, iii). These are the directions (perpendicular to cellular edges) along which cells within the monolayer are locally compressed or tensed at maximal and minimal intensity respectively. In mechanics, these directions and stresses are respectively termed principal directions and principal stresses. Principal stresses and directions can be utilised as proxies to gauge the local anisotropy of intercellular tension within the collective [49]. We observed that the maximum principal stress was mainly perpendicularly to the migrating edge of both Notch-Up and Notch-Down EC collectives (Fig. 5J). Stress anisotropy is represented by the eccentricity *ε* of an ellipse whose minor and major axes are respectively proportional to the minimum and maximum principal stresses (Fig. 5J). A lower eccentricity indicates a more isotropic stress field, with relatively uniform intercellular tension in all local directions. Conversely, a higher eccentricity indicates a more anisotropic stress field, where cells experience greater differentials of compression and/or tension along different directions. Our mechanical analyses showed that average stress anisotropy rapidly increased and then plateaued in Notch-Down EC collectives to remain approximately stable for the rest of the time window of our analyses (Fig. 5K,L). Conversely, we found that average stress anisotropy in Notch-Up EC collectives increased towards the same plateau value of Notch-Down EC collectives but at a lower rate (Fig. 5K,L). Further quantification of the average direction $\alpha$ of maximum principal stress within EC collectives (ellipse inclination $\alpha$, inset Fig. 5K) revealed that the ECs in both Notch-Up and Notch-Down EC collectives consistently demonstrated a preference for migrating along the direction of the local maximum principal stress (which also is the direction of minimum shear). Indeed, the angular distribution of maximum principal directionality (Fig. 5J) and cell speed directionality (Fig. 3F) showed similar trends predominantly aligned around the *x*-axis of directed migration (Fig. 1A). This behaviour is in line with directed collective migration of both Notch-Up and Notch-Down EC collectives via Plithotaxis [34, 48–50].

## Discussion

Directed collective cell migration is a fundamental process in development and disease [1–5], requiring individual cells to coordinate their movements while each of them acts as physical constraints on each other and so that the entire collective advances towards a predominant direction of motion. This process is believed to be a key driver of early-stage angiogenesis and is driven by the juxtacrine Notch signalling cascade in endothelial cells [4, 51–53]. During early-stage angiogenesis new blood vessels form from pre-existing ones via the collective migration of endothelial cell, which collectively leave the vessel and invade the surrounding stroma along the predominant direction of the presumptive new vessel. Here, we combined various established techniques for the micropatterning of ECM-protein on compliant substrate matrices with the quantification of cellular mechanics into a flexible in vitro framework (Figs. 1 and 2), which allowed us to study the separate and combined contributions of individual cell mechanics to the directed migration of 2D monolayers of HUVEC cells with and without juxtacrine Notch signalling. We found that (i) juxtacrine Notch signalling is dispensable for directed cell migration in 2D, as the geometry of the monolayer—with its length much greater than its width (like in vessels)—is sufficient to impart a predominant direction of motion away from the monolayer along the axis of its width (Fig. 3); (ii) Notch-Down EC collectives exhibit lower migration efficiency during collective migration, as their cellular tractions show less alignment with matrix deformations compared to Notch-Up EC collectives (Fig. 4); (iii) the direction of collective migration in HUVEC monolayers both with and without juxtacrine Notch signalling is driven by local stress anisotropy and is predominantly aligned with the direction of maximum intercellular tension (i.e., minimum intercellular shear) within the monolayer (Fig. 5), a feature considered as a signature of directed collective migration via Plithotaxis [34, 48–50]; and (iv) Notch-Up EC monolayers can expand by exerting higher tractions and sustaining higher levels of both intercellular shear and tension over longer time intervals though to a lesser extent than Notch-Down EC monolayers (Figs. 4 and 5).

The mechanisms of endothelial migration have been well studied over the past years [54]. Two characteristics of cell migration have been identified—cellular speed and its directionality—which are postulated to be regulated by different mechanisms [44, 55–58]. In recent years, increasing evidence has pointed to the critical role of cell–cell communication through Notch signalling in regulating angiogenesis [40] and cellular migration, this signalling also believed to be involved in regulating cellular migration speed of epithelial cells [59] and both speed and directionality of endothelial cells in 3D [6]. Here, we additionally observed that Notch signalling plays a predominant role in regulating 2D migration speed of HUVEC monolayers but not necessarily migration directionality. In the case of the latter, monolayer’s geometry (longer than wider, like in vessels) and local mechanics (migration in the direction of the maximum principal stress, as in Plithotaxis) showed to provide enough cues for directed collective migration of ECs in the direction of the maximum principal stress both under Notch signalling and without (Fig. 3). Notch signalling has been shown to regulate the temporal and spatial dynamics of cellular phenotype selection and rearrangement [60–63]. Endothelial cells are specified into a fast-migrating leader or slow-migrating follower phenotype through Notch signalling, where the number of leader cells increase upon inhibition of Notch signalling [64]. Notch-active collectives show increased migration speed at the edge of the cell collective, where leader cells are positioned (Fig. 3). However, upon Notch inhibition, we observed an increase in migration speed randomly distributed throughout the entire collective (Fig. 3), which is in line with leader cell migration, showing loss of leader–follower organisation.

Taken together, our mechanical analyses of 2D HUVEC monolayers, which were significantly longer than they were wide (reflecting the geometric proportions of vessels) revealed that both Notch-Up and Notch-Down EC monolayers follow the same regime of directed cell migration, both unfolding along the direction of maximum principal stress (Plithotaxis) and with non-decreasing traction forces and stress anisotropy while expanding. Several studies have elucidated the role of Plithotaxis in guiding directed cell migration along the direction of maximum principal stress, highlighting the importance of sustained traction forces and stress anisotropy in this process [24, 34, 49, 65]. These mechanical factors are crucial for maintaining coordinated migration within cell monolayers, where cells align and move cohesively under the influence of internal stress gradients. This behaviour underscores the fundamental importance of mechanical integrity in collective cell dynamics, ensuring that migration is both directed and efficient, which is critical for processes like vascular remodelling. In the Notch-Up regime, where Notch signalling unfolded undisturbedly, ECs collectively invaded the free matrix space surrounding the vessel by sustaining over a longer time interval more elevated levels of intercellular tension while progressively increasing mutual shear between cells within the collective. In the Notch-Down regime, where Notch signalling was pharmaceutically inhibited via DAPT treatment, ECs were able to undergo directed collective migration while sustaining lower levels of intercellular tension and increased shear over a shorter time interval (approximately half that of Notch-Up ECs), before both classes of monolayers would continue expanding at lower intercellular tension and shear.

For cellular migration, cells have to form cellular protrusions, attach these to the ECM by adhesion complexes, contraction of the cell body, and release rear from ECM. Essential for these steps is the remodelling of the actin cytoskeletal network into filopodia protrusions (regulated by small GTPase Cdc42), lamellipodia protrusions (regulated by Rac), and stress fibres (linked to contractile fibres by α-catenin) [66]. The actin cytoskeleton is responsible for exerting traction forces transmitted to the substrate by cell–matrix focal adhesion complexes for the generation of propulsive forces for a cell to move forward [66]. Recent research has shown that Notch signalling mediates Rho GTPase effector genes within the formation of cellular protrusions [67], actin organisation [68–70], cell–matrix focal adhesions formation [71], and its activity [72], but also cell–cell adherence [60, 69, 71, 73–75]. We observed a universal link between cellular forces, intercellular stresses and migration speed in endothelial cells during collective migration—a link that was altered within Notch-inhibited collectives (Figs. 4 and 5). Future research should reveal the exact alterations in cytoskeletal organisation as well as cell–matrix and cell–cell adhesions upon Notch-inhibition to unravel the distinct mechanisms of directed endothelial collective migration.

## Conclusions

Taken together, we developed a 2D framework to study the separate and combined contributions of individual cell mechanics to the directed migration of a collective. This framework enables users to perform high spatiotemporal analysis of migrative behaviour, cell–matrix traction forces and intercellular forces in different microenvironments. We show the applicability of this framework by studying the role of juxtacrine Notch signalling in directed collective migration of endothelial cells. Our results reveal that Notch-Up and Notch-Down HUVEC cellular monolayers adopt a distinct regime of directed collective migration via Plithotaxis. The Notch-Up regime unfolds by means of sustained and highly correlated mechanical response among cells, which is characterised by cells achieving higher directional migration speeds through increased force generation at both the cell–matrix and cell–cell interfaces. In contrast, the Notch-Down regime unfolds through a less sustained and correlated mechanical response among cells, where cellular speed differentials can be achieved without substantial changes in force generation both at the cell–matrix and cell–cell interface. Our findings point to the potential existence of a link between the juxtacrine signalling of Notch and an increased mechanical cohesiveness among cells of the collective, thus showing that this in vitro framework is a powerful approach to dissect the mechanisms of collective cell migration.

Our results indicate that Notch signalling regulates the mechanical coordination of collectively migrating endothelial cells. In Notch-Up conditions, increased mechanical cohesiveness may stabilise follower cell behaviour, while reduced force correlation in Notch-Down conditions may promote a more dynamic, leader-like phenotype. During sprouting angiogenesis, leader–follower dynamics are governed by juxtacrine Notch signalling [76], with the timing of Notch activity influencing vascular branching and network formation [77]. The distinct migration regimes we observe may reflect temporal regulation of leader cell selection [78]. Sustained coordination in Notch-Up conditions suggests that prolonged signalling reinforces follower-like behaviour and collective force transmission. In contrast, the Notch-Down regime may enhance motility and plasticity, supporting leader-like traits [79]. These behaviours are further shaped by cytoskeletal remodelling and mechanical cues [80]. Notch activation in breast cancer cells promoted actin stress fibre formation and strengthening of both cell–matrix and cell–cell adhesions resulting in increased mechanical cohesiveness and reduced cell motility [81], which supports our speculations that Notch signalling may modulate mechanical coordination through similar mechanisms in endothelial cells. Notably, Notch signalling also responds to matrix stiffness, showing increased activity on softer substrates [72]. Soft matrices also upregulate VEGFR1/2 and Dll4 via YAP-dependent pathways [82, 83], highlighting how mechanical cues influence Notch activity and endothelial function during angiogenesis.

Thus, by enabling high spatiotemporal analysis of migratory behaviour, cell–matrix traction forces and intercellular forces, our in vitro framework provides a powerful tool for dissecting the mechanistic basis of Notch-regulated collective migration. Future studies integrating this framework with 3D models and in vivo systems will be critical to further elucidate how Notch-mediated mechanical signalling shapes vascular morphogenesis in both developmental and pathological contexts.

## Methods

### Cell culture

Human Umbilical Vein Endothelial Cells (HUVECs; Lonza) were cultured in Endothelial Cell Growth Medium 2 supplemented with 0.02 ml/ml Foetal Calf Serum, 5 ng/ml Epidermal Growth Factor (recombinant human), 10 ng/ml Basic Fibroblast Growth Factor (recombinant human), 20 ng/ml Insulin-like Growth Factor (Long R3 IGF, recombinant human), 0.5 ng/ml Vascular Endothelial Growth Factor 165 (recombinant human), 1 µg/ml Ascorbic Acid, 22.5 µg/ml Heparin, 0.2 µg/ml Hydrocortisone (Promocell), and 1% penicillin/streptomycin (pen/strep, Lonza). HUVECs were cultured at 37 °C and 5% CO_2_, medium was replenished every 2–3 days and cells were passaged at a confluency of 80–90%.

### Extracellular matrix (ECM)-patterned coverslips—confined cell collective (Fig. 1B) & confined single cell (Fig. 1D)

Thirteen-millimetre glass coverslips were activated using oxygen plasma ashing with a power of 20 W for 30 s and the ashing chamber was vented with nitrogen. After plasma treatment, place the coverslips on a substrate covered with parafilm. Using a Pasteur pipet, quickly cover the complete surface of the coverslip with poly-L-lysine (PLL, 0.01%) and incubate for 30 min at room temperature. Remove the PLL solution and wash three times with 0.1 M HEPES buffer (8 < pH < 8.5). Dissolve methoxypolyethylene glycol-succinimidyl valerate (mPEG-SVA) in 0.1 M HEPES buffer (8 < pH < 8.5) at a concentration of 50 mg/ml, add a 70 µl droplet of the mPEG-SVA solution on top of the 13-mm coverslip and incubate for 1 h at room temperature. Wash the coverslip five times with PBS and store at 4 °C until usage (with a maximum of 3 days). Patterning of the coverslips was performed using ultraviolet (UV) photopatterning on a fluorescence microscope equipped with PRIMO. After calibration of the UV laser, remove the mPEG-SVA coated coverslip from the well and place it on a coverslip holder in the microscope. Add 60–70 µl photoinitiator (PLPP) on top of the coverslip and select a digital mask containing the desired pattern (black background with White strip with a width of 300 µm for collective patterning, White circle with a diameter of 35 µm for single cell patterning or completely white for single cell patterning control). UV-photopatterning was performed using a dose of 1000 mJ/mm^2^. Once finished patterning, the coverslip is removed from the holder and stored in PBS at 4 °C until usage (with a maximum of 3 days). In a cell culture hood, wash coverslip thrice in sterile PBS. Transfer patterned coverslip to a sterile dry 6-well plate (Greiner). Incubate the coverslip with 50 µl 0.2 mg/ml collagen type IV for 5 min at 4 °C. Wash the coverslips twice in PBS and once in sterile milliQ-water. Wash hard by aspirating PBS/milliQ-water on top of the coverslip to remove all collagen type IV that binds to the coverslip outside the pattern.

### Polyacrylamide (PAA) substrate preparation and ECM coating

Across the different models within the framework, PAA gels were prepared through two different methods: (Method 1) ECM-coated PAA gels (directed collective migration (Fig. 1A) & unconfined single cell (Fig. 1C)); and (Method 2) ECM-patterned PAA gels (confined cell collective (Fig. 1B) & confined single cell (Fig. 1D)).

#### (Method 1) ECM-coated PAA gels

Glass bottom 6-well plates #0 (CellVis) were treated with a bind-silane solution containing 14 × absolute ethanol (for synthesis, VWR), 1 × acetic acid (Merck) and 1 × 3-(Trimethoxysilyl)propyl methacrylate (Sigma-Aldrich). After 1 h treatment, plates were washed with absolute ethanol three times and dried using nitrogen gas. PAA gel mixture (12 kPa) was prepared containing 373.5 µl Phosphate Buffered Saline (PBS, Sigma), 93.8 µl 40% acrylamide (Bio-Rad), 25 µl 2% bis-acrylamide (Bio-Rad) and 5 µl FluoSpheres (Invitrogen), and mixed well by vortexing. Seconds before pipetting 22 µl PAA gel solution on top of the glass bottom of the plate, 2.5 µl ammonium persulfate (APS, Bio-Rad) and 0.25 µl TEMED (Sigma-Aldrich) were added to the PAA gel mixture and mixed well by vortexing. The PAA droplet was covered with an 18-mm coverslip (VWR) and left to polymerise for 1 h. After incubation, PBS was added to the wells and coverslips were gently removed using a needle and tweezer. Polymerised gels were washed twice with PBS. PAA gels were functionalised using 150 µl 1 mg/ml Sulfo-SANPAH using 365 nm wavelength ultraviolet light for 5 min. After incubation, plates were placed in a cell culture hood and washed three times 10 min with sterile PBS. Gels were coated overnight at 4 °C with 0.2 mg/ml collagen type IV (from human placenta, Sigma-Aldrich) diluted in PBS. Gels were washed twice in sterile PBS.

#### (Method 2) ECM-patterned PAA gels

In a cell culture hood, Glass bottom 12-well plates #0 (MatTek) were treated with a bind-silane solution (Method 1) for 1 h. Plates were washed with absolute ethanol and left to airdry. PAA gel mixture was prepared (Method 1) and mixed well by vortexing. Collagen type IV patterned coverslips were removed from the well, placed on plate lid rim with the collagen type IV pattern upward and left for slight airdrying. Quickly, 2.5 µl APS and 1 µl TEMED were added to the PAA gel mixture, mixed well by vortexing, and a 10 µl PAA gel droplet was pipetted in each well. Collagen type IV patterned coverslips (13 mm) were gently placed on top of the PAA gel droplets (with collagen type IV pattern facing the droplet) and gels were left to polymerise for 20 min at RT. After incubation, gels were incubated for ~ 2 h in 10 × PBS at 37 °C and coverslips were gently removed using a sterile needle and tweezer. Gels were washed twice in PBS.

### PAA hydrogel stiffness measurement

For each gel batch, one extra gel was prepared for verification of gel stiffness using nano-indentation (Optics11 Piuma Nano-Indenter). A nano-indentation tip of stiffness 0.25 N/m and tip radius 25 µm was used to perform a 7-µm indentation. The stiffness of each gel mixture is an average of 5 random independent measurements on the gel. This value was used as input within the traction force microscopy (TFM) analysis. Gel stiffness of gels in figures is 10.4 ± 0.4 kPa.

### PDMS membrane preparation—directed collective migration (Fig. 1A)

SU8-3050 (Microchem) master containing rectangular patterns of 300 µm in width, 3000 µm in length and approximate height of 50 µm was prepared using conventional soft photolithography. To create polydimethylsiloxane (PDMS) stencils with an approximate height of 15 µm, prepare fresh PDMS, degas PDMS, spincoat PDMS on master for 1 min at 3000 rpm, and cure on hotplate overnight at 85 °C. Increase temperature of hotplate to 140 °C, prepare thick borders of the PDMS stencil for handling purposes, leave to cure for 3 h at 100 °C, and cut PDMS stencils using a scalpel. Peel of the PDMS stencils from the master, wash once in 70% ethanol and store at 4 °C till usage.

### PDMS membrane passivation—directed collective migration (Fig. 1A)

Before use of PDMS stencil for cellular patterning on ECM-coated PAA gels, stencils were passivated in freshly prepared 3% Pluronic F127 (Sigma) in PBS at room temperature overnight. Right before cellular patterning in the biosafety cabinet, PDMS stencils were washed twice in sterile PBS and once in Milli-Q water and left to dry before use.

### Cell seeding

#### Directed collective migration (Fig. 1 A)

Remove PBS from well and incubate PAA gels in 500 µl medium for at least 30 min at 37 °C. Remove medium and leave the PAA gels to airdry. Using tweezers, gently place dry PDMS membrane on top of dry PAA gel. Seed a droplet of 100 µl containing 75,000 cells on top of the PDMS membrane and leave cells to attach for 2 h. Aspirate the droplet, add a 200 µl medium droplet and leave overnight. Replenish medium droplet and leave for several hours till usage (imaging). Right before imaging (1 day after seeding), PBS was added to the well and membranes were gently removed. Wells were washed twice with PBS and 3 ml medium was added.

#### Confined cell collective (Fig. 1B) and confined single cell (Fig. 1D)

Remove PBS from well, slightly airdry the PAA gel, and incubate a 50 µl droplet of medium on top of PAA gel for 30 min at 37 °C. Remove medium droplet. For confined cell collective (Fig. 1B), seed a droplet of 75 µl containing 100,000 cells on top of the PAA gel. For confined single cell (Fig. 1D), seed a droplet of 75 µl containing 7500 cells on top of the PAA gel containing Ø35 µm islands and seed a droplet of 75 µl containing 2500 cells on top of the control PAA gel (uniform patterning). Leave cells to attach for 30 min. Add 1 ml PBS to the well and wash thoroughly by aspirating on top of the cells (to ensure that there is no cell attachment outside the patterns). Wash once more with PBS. Remove PBS and add 1.5 ml medium to the well. For confined cell collective (Fig. 1B), leave cells overnight. Right before imaging (1 day after seeding), replenish medium. For confined single cell (Fig. 1D), replenish medium right before imaging (same day of seeding).

#### Unconfined single cell (Fig. 1 C)

Remove PBS from well, slightly airdry the PAA gel, and incubate a 100 µl droplet of medium on top of PAA gel for 30 min at 37 °C. Remove medium droplet, seed a droplet of 100 µl containing 2500 cells on top of the PAA gel and leave to attach for 2 h. Aspirate droplet and add 3 ml medium to the well. Right before imaging (same day of seeding), replenish medium.

### Notch signalling inhibition

DAPT, a gamma-secretase inhibitor, inhibits cleavage of the internal domain of the Notch receptor therefore prevents activation of the Notch signalling pathway. For directive collective migration (Fig. 1A) and confined cell collective (Fig. 1B), the medium droplet was aspirated after ECs attached to the ECM on the PAA gel and a fresh ECGM2 medium droplet containing DAPT dissolved in DMSO (10 µg/ml; Sigma) was incubated overnight. After removal of the PDMS stencil right before imaging, the well was filled with 3 ml of fresh ECGM2 medium containing DAPT dissolved in DMSO. For unconfined single cells (Fig. 1C), the medium droplet was aspirated and 3 ml fresh ECGM2 medium containing DAPT dissolved in DMSO (10 µg/ml) was added at least 4 h before imaging. Control wells were treated with an equivalent amount of vehicle control DMSO (2 µl/ml).

### Live cell imaging

Cells and fluomarkers were imaged using a Leica DMi8 microscope using a × 20 dry objective (0.55NA) and a 16-bit Hamamatsu Orca Flash sCMOS-camera equipped with temperature, CO2 and humidity control. The endothelial strip or single cell was placed in the centre of the field-of-view (FOV) and marked as one region-of-interest. For directed collective migration (Fig. 1A), a 3 × 1 tiled images were marked—with 10% overlap between tiles—to avoid cells migrating outside the FOV. For single cell migration modes (Fig. 1C,D), an additional zoom of 1.6 × was used to increase resolution. Time-lapse imaging was performed for approximately 15 h (overnight) with a time interval of 10 min and consisted of phase contrast imaging for visualisation of the cells and fluorescent imaging for visualisation of the fluomarkers (Fig. 2A). After time-lapse imaging, cells were removed using several droplets of 5% sodium dodecyl sulphate (Sigma) diluted in water to acquire the reference image (relaxed state of the fluomarkers).

### Image analysis

Before analysis, all timelapse images were aligned and cropped with respect to the reference image. For directed collective migration (Fig. 1A), 3 × 1 tile images were merge into one image by cross-referencing the 10% overlap of 2 neighbouring images. For each timepoint, a manual mask was created surrounding the collective or the single cell for quantitative data analysis (e.g. area) but also to only retrieve data generated by the cell(s) and extract background noise (Fig. 2B, i). Cellular velocity was analysed using Particle Image Velocimetry (PIV) on two consecutive phase contrast images of the cell(s) by diving the region-of-interest into smaller interrogation windows of 64 × 64 pixels with 0.5 overlap (Fig. 2B, ii). Displacements of the fluomarkers between any timepoint and the reference image were computed using PIV with interrogation windows of 32 × 32 pixels with 0.5 overlap (Fig. 2B, iii). Migration analysis was performed by analysing the normalised area of the mask in time and analysing the PIV migration velocity vectors (Fig. 2C, i). Cellular tractions (T; cell–matrix mechanics using traction force microscopy) were computed from the fluomarkers displacements (u) by Fourier transform-based traction microscopy of an infinite gel with a finite gel thickness using the Boussinesq equation (Fig. 2C, ii) [47]. Intercellular stresses (cell–cell mechanics using monolayer stress microscopy) were computed as a mechanical equilibrium to the forces exerted by the elastic hydrogel to the cell which arise from the traction force of the cell on the hydrogel (Fig. 2C, iii). Computations were executed in the custom 2D finite element method platform EMBRYO, a platform that was developed in the laboratory of J.J. Muñoz. We computed for each pixel a stress tensor (containing normal and shear stresses in *x*- and *y*-direction) and calculated the maximum and minimum principal stress by removal of the shear stress (Fig. 2C, iii). We computed the average normal stress (ANS; average of the maximum and minimum principal stress) as a measure of intercellular tension (positive) and compression (negative) within the collective. We computed the maximum shear stress (MSS; the difference between the principal stresses) as a measure of shear within the collective.

### Strain energy

Cell-induced deformations on a PAA hydrogel substrate can be approximated as linear elastic [84]. Our hydrogel-nanoindentation measurements confirm that the substrate has uniform material properties. Thus, the PAA behaves as a uniformly elastic sheet under the external traction force field, allowing the (total) strain energy $U$ to be computed at each time point using Continuum Mechanics through the equation

$$U=\frac{1}{2}{\int }_{A}\overrightarrow{T}\left(x,y\right)\cdot \overrightarrow{u}\left(x,y\right)\text{d}A=\frac{1}{2}{\int }_{A}|\overrightarrow{T}\left(x,y\right)|\cdot |\overrightarrow{u}\left(x,y\right)|\text{cos}\theta \left(x,y\right)\text{d}x\text{d}y.$$

Here, at any given time point $t$, term $\overrightarrow{T}\left(x,y\right)\bullet \overrightarrow{u}\left(x,y\right)$ represents the dot-product between cell traction-force vectors $\overrightarrow{T}\left(x,y\right)$ (units of Pascal, $\left[\text{Pa}\right]$) and hydrogel-substrate displacement vectors $\overrightarrow{u}\left(x,y\right)$ (units of Metres, $\left[\text{m}\right]$) in a generic point $\left(x,y\right)$ on the interface $A$ between cells and the underlying elastic substrate. $\theta \left(x,y\right)$ is the angle (units of degrees, $\left[^\circ \right]$) between vectors $\overrightarrow{T}\left(x,y\right)$ and $\overrightarrow{u}\left(x,y\right)$, whereas $\left|\overrightarrow{T}\left(x,y\right)\right|$ and $\left|\overrightarrow{u}\left(x,y\right)\right|$ represent vector magnitudes (or moduli). Term $dU=\overrightarrow{T}\left(x,y\right)\bullet \overrightarrow{u}\left(x,y\right)\text{d}x\text{d}y$ (in units of Joules, $\left[\text{J}\right]=\left[\text{Pa}\bullet \text{m}\bullet {\text{m}}^{2}\right]$) represents the (local) strain energy transferred by the cellular collective to an infinitesimal area $\text{d}A=\text{d}x\text{d}y$ around point $\left(x,y\right)$ at the cell-hydrogel interface to produce a displacement $\overrightarrow{u}\left(x,y\right)$. Consequently, $U$ is the total strain energy is the overall energy transferred or work done by the collective to the deformed elastic substrate (this energy is fully recovered when the hydrogel relaxes after cell trypsinisation and removal). Since the local strain energy density $\overrightarrow{T}\left(x,y\right)\bullet \overrightarrow{u}\left(x,y\right)$ (in units of $\left[\text{Pa}/{\text{m}}^{2}\right]=\left[\text{Pa}\bullet \text{m}\right]$) can be interpreted as the work done by the cell traction force $\overrightarrow{T}\left(x,y\right)$ to generate the substrate displacement $\overrightarrow{u}\left(x,y\right)$, the term total strain energy $U$ can also be utilised as a proxy for the total work done by the cellular collective at the cell-substrate interface $A$ to symmetrically expand via directed collective cell migration. It is important noticing that each infinitesimal contribution $dU$ to $U$ is: maximised when $\overrightarrow{T}$ and $\overrightarrow{u}$ are parallel vectors ($\theta ={0}^{\circ }$ and $\text{cos}\theta =1$, meaning the whole traction force $\overrightarrow{T}$ locally contributes to the displacement $\overrightarrow{u}$); zero when $\overrightarrow{T}$ and $\overrightarrow{u}$ are perpendicular vectors ($\theta =9{0}^{\circ }$ and $\text{cos}\theta =0$, meaning no part of traction force $\overrightarrow{T}$ locally contributes to the displacement $\overrightarrow{u}$); and minimised when $\overrightarrow{T}$ and $\overrightarrow{u}$ are antiparallel vectors ($\theta =18{0}^{\circ }$ and $\text{cos}\theta =-1$, meaning the whole traction force $\overrightarrow{T}$ locally antagonises the hydrogel’s displacement $\overrightarrow{u}$). Thus, the term $\text{cos}\theta \left(x,y\right)$ can be utilised as a metric for the local alignment between $\overrightarrow{T}$ and $\overrightarrow{u}$. In an ideally efficient system made of a cellular collective undergoing directed collective cell migration by exerting a traction force field $\overrightarrow{T}\left(x,y\right)$ parallel to the displacement field $\overrightarrow{u}\left(x,y\right)$—i.e. $\theta \left(x,y\right)={0}^{\circ }$ and $\text{cos}\theta \left(x,y\right)=1$ everywhere in $A$—there would be full local alignment between $\overrightarrow{T}$ and $\overrightarrow{u}$ everywhere in $A$ and total strain energy would be the maximum possible and equal to:

$${U}^{*}=\frac{1}{2}\int \left|\overrightarrow{T}\left(x,y\right)\right|\bullet \left|\overrightarrow{u}\left(x,y\right)\right|\text{d}x\text{d}y.$$

This allows to define a metric $\eta$ for total efficiency of directed collective migration as:

$$\eta =\frac{U}{{U}^{*}}=\frac{\int \left|\overrightarrow{T}\left(x,y\right)\right|\bullet \left|\overrightarrow{u}\left(x,y\right)\right|\text{cos}\theta \left(x,y\right)\text{d}x\text{d}y}{\int \left|\overrightarrow{T}\left(x,y\right)\right|\bullet \left|\overrightarrow{u}\left(x,y\right)\right|\text{d}x\text{d}y}.$$

The value of $\eta$ must vary between 0 and 1 by definition. The highest value $\eta =1$ indicates perfectly directed collective cell migration, where all cellular traction forces $\overrightarrow{T}\left(x,y\right)$ translate in propelling a cell movement in the direction opposite to the hydrogel displacement $\overrightarrow{T}\left(x,y\right)$. Lower values of $\eta$ indicate less efficient migration, with traction forces locally acting in less coordinated ways or even in directions opposing directed motion. It is worth stressing that, in the light of the definition $\eta$, the term $\text{cos}\theta \left(x,y\right)$ can also be intrinsically utilised as a metric for local efficiency of directed collective migration.

### Post-processing methods

Spatio- and temporal post-processing analysis methods were performed on magnitude of the migration velocity vector (speed), traction vector (traction magnitude) or principal stresses. To analyse a certain quantity in time (Fig. 2D, i), we plot a certain quantity on the *y*-axis and time on the *x*-axis. For each timepoint, we compute the median value of a certain quantity within the mask and average the median values of multiple regions-of-interest. To analyse the distribution of a certain quantity (Fig. 2D, ii), we plot a certain quantity on the *y*-axis and certain regions (e.g. centre vs edge) on the *x*-axis. For each timepoint, we divide the mask into regions of approximately equal amount of pixels. This is achieved by reducing the mask size of the cell collective by 1-pixel increments from the edge of the mask in the direction of migration, until the remaining central zone contains fewer than half of the total pixels of the original mask. At *t* = 0 h, the mask is, on average, 8322 pixels in size, with each zone representing approximately 50% ± 0.7% of the total pixel count. A difference of 0.7% between the sizes of the central and edge zones is considered negligible and assumed to have no significant effect on our findings. We compute the median value of a certain quantity within this region and average this in time. Each region-of-interest is represented as one value. This analysis can be extended by additional temporal analysis, by averaging for specific timepoints. For spatiotemporal visualisation of a certain quantity, we compute a kymograph (Fig. 2D, iii). We erode the mask in vertical single lines (1-pixel in width) from the edge to the centre of the mask. For each timepoint, across each vertical line, we compute the median value of a certain quantity resulting in a single horizontal line per timepoint showing spatial information of a certain quantity. By stacking these horizontal lines of each timepoint below one another, we visualised temporal information of a certain quantity. For visualisation purposes, only a representative half kymograph is shown. Directionality analysis was performed by computing—for each pixel—the angle between the *x*-axis and the migration velocity or traction angle or maximum principal stress (Fig. 2D, iv). For the migration velocity and traction vector (pointing in one direction), this angle varies from − 180–180°, whereas for the maximum principal stress, this angle varies from 0 to 180° (dual direction). For visualisation purposes, the migration velocity and traction vectors were displayed in absolute values meaning that the negative angles are flipped along the *y*-axis within the diagram. This allows visualisation of two diagrams within one figure panel. Additional anisotropy of the stress was computed by plotting an ellipse on top of the principal stresses and calculated the eccentricity *e* of the ellipse using the following equation: $e=\sqrt{{}^{{{\sigma }_{min}}^{2}}\!\left/ \!{}_{{{\sigma }_{max}}^{2}}\right.}$. An eccentricity close to 0 resembles a circle (where the maximum principal stress is equal to the minimum principal stress), whereas an eccentricity close to 1 resembles a narrow ellipse (where the maximum principal stress is much greater compared to the minimum principal stress).

### Statistical analysis

For single cell analysis, normality of the distribution was tested using a Shapiro–Wilk test. In case of normality, an unpaired *t*-test between two groups was performed. In case of no normality, a Mann–Whitney test between two groups was performed. Due to low sampling within the collective analysis, no statistical analysis was performed but fold-changes were mentioned.

## Supplementary Information

Additional file 1: Supplementary results. Fig. S1. Schematic overview of experimental procedures. Fig. S2. Additional data migration analysis. Fig. S3. Additional data Traction Force Microscopy.

Additional file 2: Video S1. Endothelial cells undergoing directed collective migration where juxtacrine Notch signalling is active. Timestamp and scalebar are displayed. Video S2. Endothelial cells undergoing directed collective migration where juxtacrine Notch signalling is inhibited. Timestamp and scalebar are displayed.

Additional file 3: Video S3. Confined endothelial cell collective where juxtacrine Notch signalling is active. Timestamp and scalebar are displayed. Video S4. Confined endothelial cell collective where juxtacrine Notch signalling is inhibited. Timestamp and scalebar are displayed.

Additional file 4: Video S5. Confined single endothelial cell through island ECM patterning. Juxtacrine Notch signalling is active. Timestamp and scalebar are displayed. Video S6. Free single endothelial cell on ECM patterned substrate as technical control of the confinement technique. Juxtacrine Notch signalling is active. Timestamp and scalebar are displayed.

Additional file 5: Video S7. Free single endothelial cell using the unconfined technique. Juxtacrine Notch signalling is active. Timestamp and scalebar are displayed. Video S8. Free single endothelial cell using the unconfined technique. Juxtacrine Notch signalling is inhibited. Timestamp and scalebar are displayed.

## Data Availability

All data generated or analysed in this study are included in this published article and its supplementary information files or are available from the corresponding author upon reasonable request. The data analysis methods are detailed in the Methods section, and the code is available upon reasonable request.
