# Supplementary material for: An integrated 2D framework for quantifying cellular mechanics reveals the impact of juxtacrine Notch signalling on directed collective migration of endothelial cells
Source: BMC Biol. 2025 Oct 1;23:291. doi: 10.1186/s12915-025-02396-4 (PMC12486679; doi:10.1186/s12915-025-02396-4)
Supplement: Supplementary file 1 — Additional file 1: Supplementary results. Fig. S1. Schematic overview of experimental procedures. Fig. S2. Additional data migration analysis. Fig. S3. Additional data Traction Force Microscopy. [file 12915_2025_2396_MOESM1_ESM.pdf]

Additional file 1. Supplementary results (1/3).

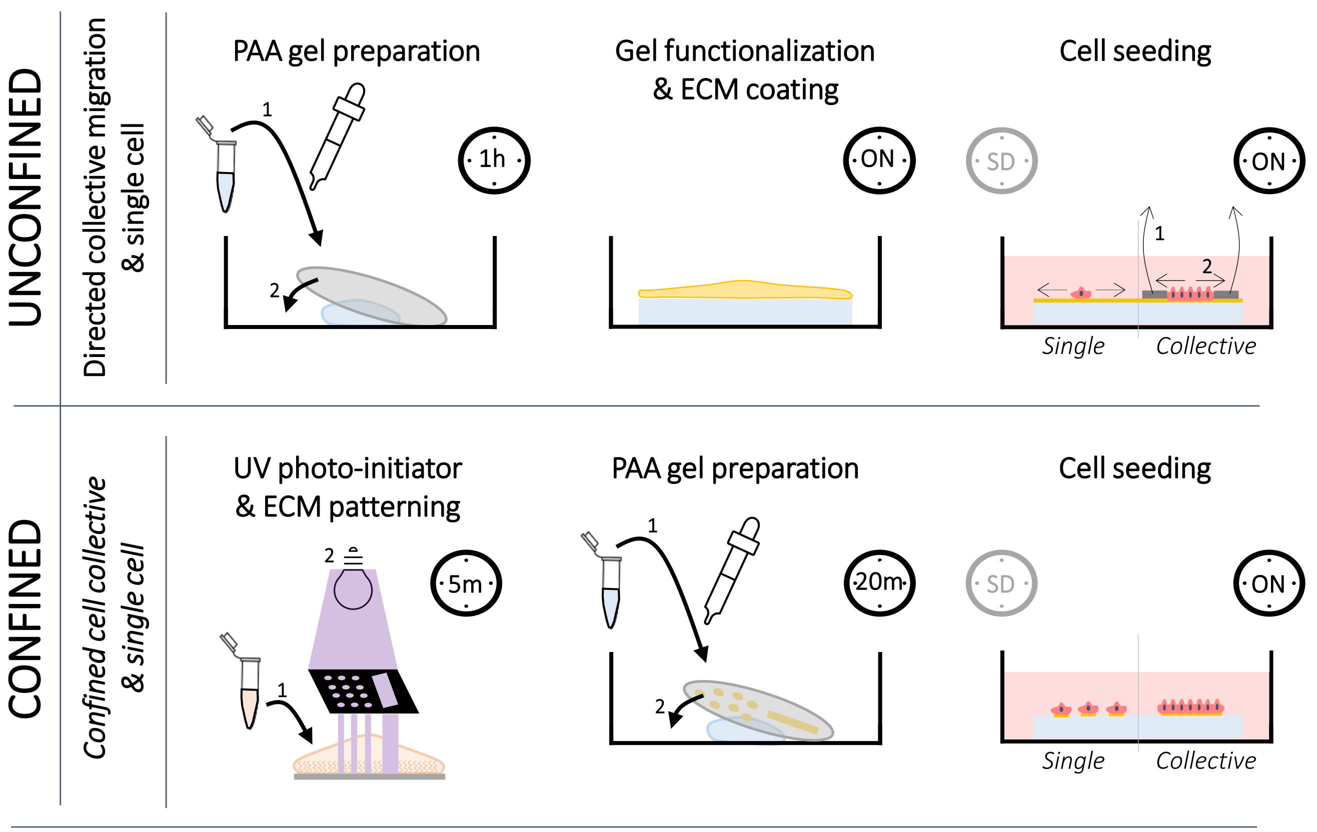

Fig S1. Schematic overview of experimental procedures. Unconfined experimental procedure (top row) starts with pipetting a droplet of polyacrylamide (PAA) gel mix (1) which is covered with a glass coverslip (2). After 1 hour of polymerization, the coverslip is removed and the PAA gel functionalized and coated overnight (ON) with an ECM-protein of interest. For single cell analysis, a low density of cells is seeded on top of the gel and imaging was performed on the same day (SD). For directed collective migration, a high density of cells is seeded on top of a PDMS stencil (with a rectangular opening) on top of the gel. Cells were left in the stencil overnight to reach confluency. Upon removal of the PDMS stencil (1), cells start to migrate (2), and imaging was performed. Confined experimental procedure (bottom row) start with UV-photo initiator patterning on a coverslip. ECM was patterned by coating the patterned coverslip for 5 minutes. In the meantime, PAA gel mix was prepared. The ECM-patterned coverslip was placed on top of a droplet PAA gel mix. After 20 minutes of polymerization, the coverslip was removed. For single cell analysis, a low density of cells is seeded on top of the gel, washed roughly with PBS after 30 minutes and images was performed on the same day. For confined cell collective, a high density of cells was seeded, washed roughly with PBS after 30 minutes and left overnight to reach confluency. Medium was refreshed and imaging was performed.

Additional file 1. Supplementary results (2/3).

Confined cell collective

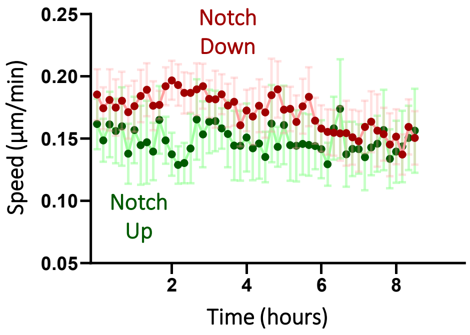

Fig S2. Additional data migration analysis. Migration speed analysis in time of the confined cell collective (Fig. 1B) based on PIV analysis of the cells. Each dot represents one timepoint, which is an average value over multiple positions. Notch-Up represents collectives treated with DMSO; Notch-Down represents collectives treated with DAPT. Data is represented as mean ± SEM.

Additional file 1. Supplementary results (3/3).

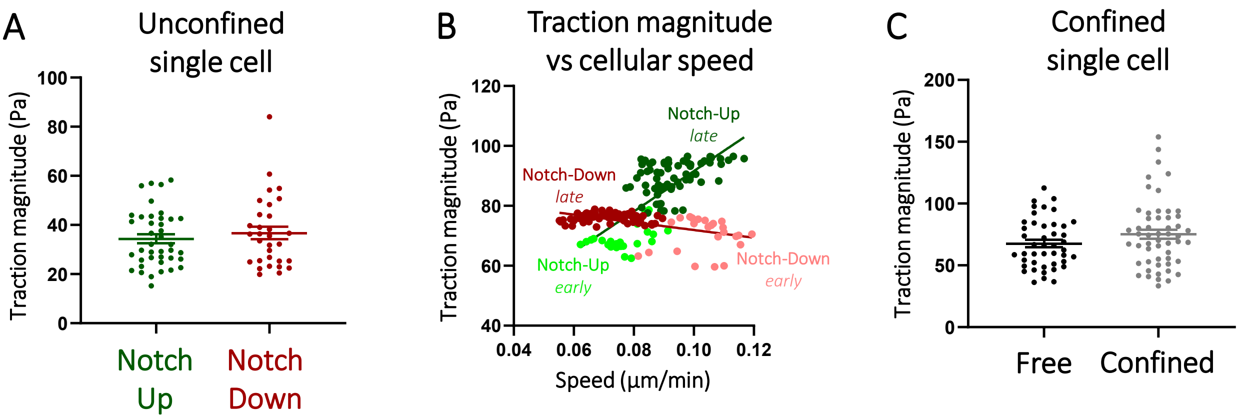

Fig S3. Additional data Traction Force Microscopy. (A) Traction force magnitude analysis of unconfined single cells (Fig. 1C). Each dot represents one single cell, which is an average value over time. Shapiro-Wilk normality was tested (no normal distribution; $p<0.05$) and a Mann-Whitney test was performed (ns; $p=0.6825$). (B) Correlation of traction magnitude and cellular speed during directed collective migration, during early phase of migration (t<4 hours; light color) and late phase of migration (t>4 hours; dark color). Each dot represents one timepoints, which is an average value over multiple positions. Fitted line by linear regression (Notch-Up: $y=637.5x+24.2$ ($R^2=0.54$); Notch-Down: $y=-128.9x+84.8$ ($R^2=0.29$)). (C) Traction force magnitude analysis of confined single cells (Fig. 1D) compared to its control free single cells (free confined single cell represents a single cell that is free to migrate on top of a very large pattern of ECM which is performed as control of the technique). Each dot represents one single cell, which is an average value over time. Shapiro-Wilk normality was tested (no normal distribution; $p<0.05$) and a Mann-Whitney test was performed (ns; $p=0.2642$). Notch-Up represents collectives treated with DMSO; Notch-Down represents collectives treated with DAPT. Data is represented as mean ± SEM.
